# Temporal changes in the epidemiology, management, and outcome from acute respiratory distress syndrome in European intensive care units: a comparison of two large cohorts

**DOI:** 10.1186/s13054-020-03455-8

**Published:** 2021-02-25

**Authors:** Yasser Sakr, Bruno François, Jordi Solé-Violan, Katarzyna Kotfis, Ulrich Jaschinski, Angel Estella, Marc Leone, Stephan M. Jakob, Xavier Wittebole, Luis E. Fontes, Miguel de Melo Gurgel, Thais Midega, Jean-Louis Vincent, V. Marco Ranieri, the SOAP and ICON Investigators, Georg Delle Karth, Volker Draxler, Gottfried Filzwieser, Werner Heindl, Gerhard Kellner, Thomas Bauer, Kurt Lenz, Edmund Rossmann, Christian Wiedermann, Patrick Biston, Didier Chochrad, Vincent Collin, Pierre Damas, Johan Decruyenaere, Eric Hoste, Jacques Devriendt, Benoît Espeel, Vincent Fraipont, Etienne Installe, Manu Malbrain, Guy Nollet, Jean-Charles Preiser, Jan Raemaekers, Alain Roman, Marc Simon, Herbert Spapen, Walter Swinnen, Frédéreic Vallot, Jean-Louis Vincent, Ivan Chytra, Lukas Dadak, Ivan Herold, Ferdinand Polak, Martin Sterba, Morten Bestle, Kurt Espersen, Henrik Guldager, Karen-Liese Welling, Dag Nyman, Esko Ruokonen, Kari Saarinen, Djillali Annane, Philippe Catogni, Gabriel Colas, François Coulomb, Rene Dorne, Maite Garrouste, Christian Isetta, Jérôme Larché, Jean-Roger Le Gall, Henry Lessire, Yannick Malledant, Philippe Mateu, Michel Ossart, Didier Payen, Pascal Schlossmacher, Jean-François Timsit, Stéphane Winnock, Jean-Pierre Sollet, Laurent Mallet, Peter Maurer, Jean-Michel Sab, Gueclue Aykut, Frank Brunkhorst, Axel Nierhaus, Michael Lauterbach, Max Ragaller, Rainer Gatz, Herwig Gerlach, Deitrich Henzler, Hand-Bernd Hopf, Hilmar Hueneburg, Waheed Karzai, Ansgar Keller, Uwe Kuhlmann, Julia Langgartner, Cornelia Manhold, B. Reith, Tobias Schuerholz, Claudia Spies, Reimund Stögbauer, J. Unterburger, Phyllis-Maria Clouva-Molyvdas, George Giokas, Eleni Ioannidou, Alexandra Lahana, Alexandros Liolios, Katerina Marathias, George Nakos, Antonia Tasiou, Hercules Tsangaris, Peter Tamasi, Brian Marsh, Michael Power, Charles Sprung, Bonizella Biagioli, Franco Bobbio Pallavicini, Antonio Pesenti, Carlo Capra, Francesco Della Corte, Pierpaulo Donadio, Abele Donati, Antonino Giarratano, Tulli Giorgio, Daniela Giudici, Stefano Greco, Alberto Guadagnucci, Gaetano Lapichino, Sergio Livigni, Gabriella Moise, Giuseppe Nardi, Eltore Panascia, M. Pizzamiglio, V. M. Ranieri, Ranieri Rosi, Alberto Sicignano, Maurizio Solca, G. Vignali, Italo Volpe Rinonapoli, Michel Barnas, Ernst E. De Bel, Anne-Cornelie De Pont, Johan Groeneveld, Maarten Nijsten, Liang-Hai Sie, Durk F. Zandstra, Svein Harboe, Svante Lindén, Renata Z. Lovstad, Harald Moen, Nils Smith-Erichsen, Andrjej Piotrowski, Ewa Karpel, Eduardo Almeida, Rui Moreno, Antonio Pais-De-Lacerda, Jose A. Paiva, Isabel Serra, Jorge M. Pimentel, Daniela Filipescu, Krsta Jovanovic, Peter Malik, Kosec Lucka, Gorazd Voga, Cesar Aldecoa Alvarez-Santullano, Antonio Artigas, Elizabeth Zavala, Angels Escorsell, Jose Nicolas, José JIzura Cea, Luis Marina, Juan Montejo, Eduardo Palencia, Francisco Santos, Rafael Sierra-Camerino, Fernando Sipmann, Keld Brodersen, Jan Haggqvist, Dan Hermansson, Hans Hjelmqvist, Kuno Heer, Giorgio Loderer, Marco Maggiorini, Hervé Zender, Peter Andrews, Balraj Appadu, Casiano Barrera Groba, Jeremy Bewley, Ken Burchett, Philip Chambers, John Coakley, Doris Doberenz, Nigel Eastwood, Andrew Ferguson, Jonathan Fielden, Jacqueline Gedney, Kevin Gunning, David Harling, Stas Jankowski, David Jayson, Andrew Kilner, Venketachalam Krishna-Kumar, K. Lei, S. Mackenzie, Peter Macnaughton, Gernot Marx, C. McCulloch, Paul Morgan, Andrew Rhodes, Chris Roberts, Mark Russell, Darell Tupper-Carey, Maggie Wright, Linda Twohey, James Watts, Rae Webster, Dewi Williams, Philipp Urbanek, Joachim Schlieber, Johann Reisinger, Johann Auer, Andreas Hartjes, Andreas Lerche, Thomas Janous, Eveline Kink, Walter Krahulec, Karl-Heinz Smolle, Marc Van Der Schueren, Patrick Thibo, Marc Vanhoof, Ibis Ahmet, Philippe Gadisseux, Philippe Dufaye, Olivier Jacobs, Alain Dive, Yves Bouckaert, Eric Gilbert, Benjamin Gressens, Eric Pinck, Jan J. De Waele, Rocio Rimachi, Dan Gusu, Koen De Decker, Kakisidi Mandianga, Luc Heytens, Xavier Wittebole, Olivier Van Collie, Wout Vandenheede, Peter Rogiers, Pavel Pavlik, Jan Manak, Eva Kieslichova, Radovan Turek, Michal Fischer, Radka Valkova, Pavel Dostal, Jan Malaska, Roman Hajek, Alexandra Židková, Pavel Lavicka, Piotr Kolodzeike, Mary Kruse, Torben Andersen, Veli-Pekka Harjola, Marc Leone, Alain Durocher, Serge Moulront, Alain Lepape, Marie-Reine Losser, Philippe Cabaret, Evangelos Kalaitzis, Elie Zogheib, Philippe Charve, Bruno François, Jean-Yves Lefrant, Bassam Beilouny, Xavier Forceville, Benoit Misset, Frederic Jacobs, Bernard Floccard, Alain Wynckel, Vincent Castelain, Alexandre Faure, Pierre Lavagne, Thierry Lepoivre, Mouhamed D. Moussa, Antoine Vieillard-Baron, Michel Durand, Marc Gainnier, Carole Ichai, Stefan Arens, Clemens Hoffmann, Magnus Kaffarnik, Claus-Jorg Scharnofske, Ingo Voigt, Claus Peckelsen, Matthias Weber, Jochen Gille, Andreas Lange, Georg Schoser, Armin Sablotzki, Ulrich Jaschinski, Andreas Bluethgen, Frank Vogel, Andreas Tscheu, Thomas Fuchs, Michael Wattenberg, Torsten Helmes, Stefan Scieszka, Matthias Heintz, Samir Sakka, Johannes Kohler, Fritz Fiedler, Matthias Danz, Yasser Sakr, Reimer Riessen, Thomas Kerz, Alexander Kersten, Frank Tacke, Gernot Marx, Thomas Volkert, Axel Schmutz, Stefan Kluge, Peter Abel, Rolf A. Janosi, Stefan Utzolino, Hendrik Bracht, Susanne Toussaint, Maria Giannakou Peftoulidou, Pavlos Myrianthefs, Apostolos Armaganidis, Christina Routsi, Angela Xini, Eleni Mouloudi, Ioannis Kokoris, George Kyriazopoulos, Sawas Vlachos, Athena Lavrentieva, Panagio Partala, Laszlo Medve, Agnes Sarkany, Ildiko Kremer, Zsuzsa Marjanek, Joan Barry, Ruth A. O’Leary, Catherine Motherway, Mohammad Faheem, Eimhin Dunne, Maria Donnelly, Torsten Konrad, Jonathan Cohen, Oded Sold, Eleonora Bonora, Carola Achilli, Sandra Rossi, Giacomo Castiglione, Adriano Peris, Daniela Albanese, Nino Stocchetti, Giuseppe Citerio, Lorella Mozzoni, Erminio Sisillo, Pasquale De Negri, Monica Savioli, Pietro Vecchiarelli, Florin Puflea, Vladimir Stankovic, Giulio Minoja, Silvia Montibeller, Plinio Calligaro, Raffaella Sorrentino, Marco Feri, Massimo Zambon, Elena Colombaroli, Tommaso Pellis, Massimo Antonelli, Antonino Gullo, Cosimo Chelazzi, Antonella De Capraris, Nicolo Patroniti, Massimo Girardis, Frederico Franchi, Giorgio Berlot, Hubert Ponssen, Julia Ten Cate, Laura Bormans, Satria Husada, Marc Buise, Ben Van Der Hoven, Auke Reidinga, Michael Kuiper, Peter Pickkers, Georg Kluge, Sylvia Den Boer, Jozef Kesecioglu, Henk Van Leeuwen, Hans Flaatten, Skule Mo, Julia Kolbusz, Andrzej Kübler, Beata Mielczarek, Malgorzata Mikaszewska-Sokolewicz, Katarzyna Kotfis, Barbara Tamowicz, Wiktor Sulkowski, Piotr Smuszkiewicz, Andrzej Pihowicz, Ewa Trejnowska, Vitor Branco, Fernando Rua, Estevao Lafuente, Marta Sousa, Nuno Catorze, Maria Barros, Luis Pereira, Ana Vintém De Oliveira, Jose Gomes, Isable Gaspar, Maria F. Pereira, Maria Cymbron, Antonio Dias, Sofia Beirao, Rosa Ribeiro, Pedro Povoa, Filomena Faria, Zelia Costa-E-Silva, Jose J. Nóbrega, Fatima Fernandes, Natalia Hagau, Gabriela Droc, Mary Nicoleta Lupu, Alexandru Nica, Radu Stoica, Dana Rodica Tomescu, Dacia Laurentia Constantinescu, Georgica MValcoreanu Zbaganu, Adriana Slavcovici, Ljiljana Soskic, Ivan Palibrk, Radmilo Jankovic, Bojan Jovanovic, Milena Pandurovic, Vesna Bumbasirevic, Boris Uljarevic, Maja Surbatovic, Nebojsa Ladjevic, Garri Slobodianiuk, Viliam Sobona, Andrea Cikova, Andrea Gebhardtova, Erik Rupnik, Lucka Kosec, Milena Kerin Povšic, Irena Osojnik, Viktorija Tomic, Javier González, Jesus Pérez Valenzuela, Pablo Vidal-Cortés, Pilar Posada, Ignacio Martin-Loeches, Noelia Muñoz Guillén, Mercedes Palomar, Jordi Sole-Violan, Antoni Torres, Miguel AGonzalez Gallego, Gerardo Aguilar, Raquel Montoiro Allué, Monica Argüeso, Martin Parejo, Manuel Palomo Navarro, Anton Jose, Nicholas Nin, Francisco Alvarez Lerma, Oscar Martinez, Eva Tenza Lozano, Sara Arenal López, Maria JPerez Granda, Salvador Moreno, Clara Llubia, Carmen De La Fuente Martos, Paloma Gonzalez-Arenas, Noemi Llamas Fernández, Bernard Gil Rueda, Isabel Estruch Pons, Nieves Cruza, Fernando Maroto, Angel Estella, Ana Ferrer, Lisardo Iglesias Fraile, Brigida Quindos, Amaia Quintano, Maria T. Tebar, Pablo Cardinal, Antonio Reyes, Alejandro Rodríguez, Ana Abella, Santiago García Del Valle, Santiago Yus, Emilio Maseda, Jose A. Berezo, Armando Tejero Pedregosa, Clara Laplaza, Ricard Ferrer, Jesus Rico-Feijoo, Marina Rodríguez, Pablo Monedero, Karin Eriksson, David Chabanel, Bernd Frankenberger, Stephan Jakob, Shiju Matthew, Robert Downes, Andrew Johnston, Roseanne Meacher, Rick Keays, Philip Haji-Michael, Chris Tyler, Simon Jones, David Tyl, Andrew Ball, John Vogel, Malcolm Booth, Paul Downie, Malcolm Watters, Stephen Brett, Marc Garfield, Lynn Everett, Sarah Heenen, Sandeep Dhir, Zoe Beardow, Marthinus Mostert, Steve Brosnan, Nuno Pinto, Stephen Harris, Andy Summors, Andrew Norton, Alastair Rose, Rebecca Appelboam, Omubo Davies, Emma Vickers, Banwari Agarwal, Tamas Szakmany, Stephen Wimbush, Ingeborg Welters, Rupert Pearse, Robin Hollands, Justin Kirk-Bayley, Nick Fletcher, Barbara Bray, David Brealey, Peter Alexander, Steven Henderson, Chris Hargreaves, Heather Black, Kiran Gowda

**Affiliations:** 1 Department of Anaesthesiology and Intensive Care, Uniklinikum Jena, Jena, Germany; 2 Intensive Care Unit and Inserm CIC 1435 & UMR 1092, Dupuytren University Hospital, Limoges, France; 3 Department of Intensive Care, Hospital Universitario de Gran Canaria Dr. Negrín, Las Palmas de Gran Canaria, Spain; 4 Department of Anesthesiology, Intensive Therapy and Acute Intoxications, Pomeranian Medical University, Szczecin, Poland; 5 Klinik für Anästhesiologie und Operative Intensivmedizin, Universitätsklinik Augsburg, Universität Augsburg, Augsburg, Germany; 6 Intensive Care Unit, Hospital SAS Jerez, Jerez, Spain; 7 Service d’Anesthésie et de Réanimation, APHM, Hôpital Nord, Aix Marseille Université, Marseille, France; 8 Department of Intensive Care Medicine, University Hospital Bern, University of Bern, Bern, Switzerland; 9 Department of Critical Care, Cliniques Universitaires St Luc, UCLouvain, Brussels, Belgium; 10 Departamento de Medicina Baseada em Evidências, Medicina Intensiva, Urgência e Emergência - Faculdade de Medicina de Petrópolis, Petrópolis, Brazil; 11 Department of Critical Care Medicine, Hospital Israelita Albert Einstein, São Paulo, Brazil; 12 Department of Intensive Care, Erasme University Hospital, Université Libre de Bruxelles, Route de Lennik, 808, 1070 Brussels, Belgium; 13 Department of Medical and Surgical Science, Anesthesia and Intensive Care, Policlinico di Sant’Orsola, Alma Mater, University of Bologna, Bologna, Italy

**Keywords:** Respiratory failure, ARDS, Airway pressures, Driving pressure, Tidal volume, Mechanical ventilation

## Abstract

**Background:**

Mortality rates for patients with ARDS remain high. We assessed temporal changes in the epidemiology and management of ARDS patients requiring invasive mechanical ventilation in European ICUs. We also investigated the association between ventilatory settings and outcome in these patients.

**Methods:**

This was a post hoc analysis of two cohorts of adult ICU patients admitted between May 1–15, 2002 (SOAP study, *n* = 3147), and May 8–18, 2012 (ICON audit, *n* = 4601 admitted to ICUs in the same 24 countries as the SOAP study). ARDS was defined retrospectively using the Berlin definitions. Values of tidal volume, PEEP, plateau pressure, and FiO_2_ corresponding to the most abnormal value of arterial PO_2_ were recorded prospectively every 24 h. In both studies, patients were followed for outcome until death, hospital discharge or for 60 days.

**Results:**

The frequency of ARDS requiring mechanical ventilation during the ICU stay was similar in SOAP and ICON (327[10.4%] vs. 494[10.7%], *p* = 0.793). The diagnosis of ARDS was established at a median of 3 (IQ: 1–7) days after admission in SOAP and 2 (1–6) days in ICON. Within 24 h of diagnosis, ARDS was mild in 244 (29.7%), moderate in 388 (47.3%), and severe in 189 (23.0%) patients. In patients with ARDS, tidal volumes were lower in the later (ICON) than in the earlier (SOAP) cohort. Plateau and driving pressures were also lower in ICON than in SOAP. ICU (134[41.1%] vs 179[36.9%]) and hospital (151[46.2%] vs 212[44.4%]) mortality rates in patients with ARDS were similar in SOAP and ICON. High plateau pressure (> 29 cmH_2_O) and driving pressure (> 14 cmH_2_O) on the first day of mechanical ventilation but not tidal volume (> 8 ml/kg predicted body weight [PBW]) were independently associated with a higher risk of in-hospital death.

**Conclusion:**

The frequency of and outcome from ARDS remained relatively stable between 2002 and 2012. Plateau pressure > 29 cmH_2_O and driving pressure > 14 cmH_2_O on the first day of mechanical ventilation but not tidal volume > 8 ml/kg PBW were independently associated with a higher risk of death. These data highlight the continued burden of ARDS and provide hypothesis-generating data for the design of future studies.

## Introduction

The first formal description of acute respiratory distress syndrome (ARDS) dates back to 1967 [1]; however, it was only in 1994 that a broad consensus to define this complex syndrome was achieved [2]. These definitions were widely adopted by clinicians and researchers over the subsequent two decades. In 2012, however, a new definition of ARDS, the Berlin definition, was developed to address some of the limitations of the earlier definition [3].

Several aspects related to the management of patients with ARDS have changed over the last few decades, including use of lung protective ventilation [4], prone positioning [5], and extracorporeal membrane oxygenation (ECMO) [6, 7]. Despite these changes in patient management and respiratory support, ARDS is still associated with mortality rates between 40 and 60% and represents a high burden on intensive care resources [8]. Although several studies have assessed the epidemiology of, outcome from, and patterns of respiratory support in patients with ARDS [8–13], temporal changes have not been widely reported [14, 15] because of the use of different definitions and the considerable heterogeneity among cohorts. However, assessment of these changes is important to understand the evolution of the burden of the disease overtime and to trace the effects of possible changes in clinical practice.

Importantly, mechanical ventilation, the main pillar in the management of patients with ARDS, has been recognized as a possible cause of lung damage or ventilator-induced lung injury (VILI), which may have a negative impact on outcome [16, 17]. Accumulating evidence suggests that adopting a lung-protective strategy [4], by implementing low tidal volume, low plateau pressure, and titrated positive end-expiratory pressure (PEEP), does not per se preclude the development of VILI [18–21]. Assessment of the possible impact of ventilatory parameters on outcome may help in developing new approaches that may minimize VILI and improve survival.

In this post-hoc analysis, we tested the hypothesis that management of ARDS would change over time, especially with respect to ventilator settings, including driving pressure, which would have an impact on outcome in patients with ARDS. We therefore first assessed temporal changes in the epidemiology and management of ARDS requiring mechanical ventilation in European intensive care units (ICUs) included in two large observational studies, performed in 2002 (SOAP study) [22] and 2012 (ICON audit) [23], and second investigated the possible association between ventilatory settings on the first day of ARDS and outcome.

## Methods

**Fig. 1:**
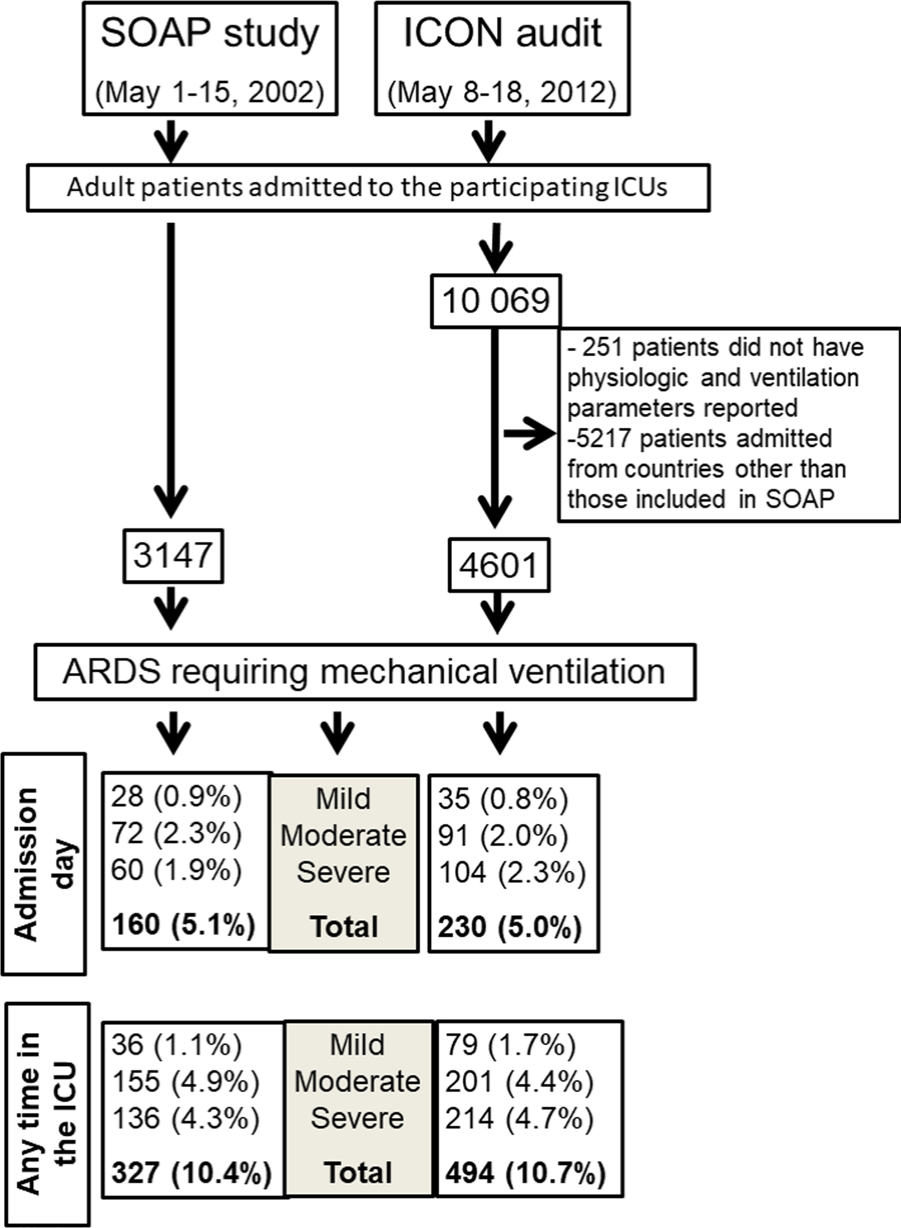
Flow diagram showing patient inclusion

This was a post hoc analysis of two multicenter European cohorts. The SOAP study was conducted in 24 European countries and included 3147 patients [22]. The ICON audit included 10,069 patients from 82 countries worldwide [23]. For the purposes of this comparison, we considered only the 4601 ICON patients who were admitted to ICUs in the same 24 European countries as in the SOAP study and had physiologic and ventilation data recorded in the ICU (Fig. 1, Additional file 1: Table S1). For both studies, recruitment for participation was by open invitation and participation was voluntary. Institutional review board approval for both studies was obtained by the participating institutions according to local ethical regulations.

Participating ICUs (see Additional file 1: e-Appendix) were asked to prospectively collect data on all adult patients admitted between May 1 and 15, 2002, for the SOAP study and between May 8 and 18, 2012, for the ICON audit. In both studies, patients who stayed in the ICU for < 24 h for routine postoperative surveillance were not included. Re-admissions of previously included patients were also not included.

### Data collection

Data were collected daily during the ICU stay for a maximum of 28 days. Data collection on admission included demographic data and comorbid diseases as well as source and reason for admission. Clinical and laboratory data for the Simplified Acute Physiology Score II (SAPS II) [24] score were recorded as the worst values within 24 h after admission. A daily evaluation of organ dysfunction/failure (cardiovascular, respiratory, renal, hepatic, coagulation, and central nervous systems) was performed using the sequential organ failure assessment (SOFA) score [25].

Values of tidal volume, PEEP, plateau pressure (Pplat) and fraction of inspired oxygen (FiO_2_) corresponding to the most abnormal value of arterial PO_2_ (PaO_2_) or arterial O_2_ saturation (SaO_2_) were recorded every 24 h; the mode of mechanical ventilation was not recorded. Patients were followed up for outcome data until death, hospital discharge or for 60 days.

### Definitions

Patients were retrospectively identified as having ARDS requiring mechanical ventilation if they presented all the following: (a) severe hypoxemia, as defined by a PaO_2_/FiO_2_ ratio < 300 mmHg with a minimum of 5 cmH_2_O PEEP; (b) presence of bilateral lung infiltrates on the chest radiograph; (c) no evidence of pre-existing heart failure; (d) absence of chronic obstructive pulmonary disease (COPD) or other chronic pulmonary disorders; (e) invasive mechanical ventilation. The severity of ARDS was categorized according to the Berlin definitions into mild, moderate, and severe [6].

For calculation of tidal volume per predicted body weight (PBW), the average PBW of male patients was calculated as equal to 50 + [0.91 (height in centimeters—152.4)]; and that of female patients as equal to 45.5 + [0.91 (height in centimeters—152.4)] [4]. We calculated driving pressure as the difference between Pplat and PEEP. Due to the observational nature of the original studies [22, 23], the management of ARDS did not follow a predefined protocol.

Non-respiratory organ failure was defined as a SOFA score > 2 for the organ in question.

### Outcome parameters

The primary outcome parameter was in-hospital mortality within 60 days of admission to the ICU. Secondary outcome parameters included death in the ICU, ICU and hospital lengths of stay, and organ failure as assessed by the SOFA score.

### Statistical analysis

All data were processed and analyzed in the Department of Intensive Care of Erasme Hospital, University of Brussels, in collaboration with Jena University Hospital, Jena, Germany. Data were analyzed using IBM® SPSS® Statistics software, v.21 for Windows (IBM, Somers, NY, USA). Data were reviewed for plausibility and availability of the outcome parameter, and any doubts were clarified with the center in question. There was no on-site monitoring. Missing data represented < 6% of the data collected for SOAP and 6.1% of the ICON data.

Data are summarized using means with standard deviation, medians and interquartile ranges, or numbers and percentages. Difference testing between groups was performed using Student’s t test, Mann–Whitney test, Chi–square test or Fisher’s exact test, as appropriate. The Kolmogorov–Smirnov test was used, and histograms and quantile–quantile plots were examined to verify whether there were significant deviations from the normality assumption of continuous variables.

To evaluate the possible association between ventilatory parameters and outcome in patients with ARDS, we grouped the patients with ARDS from the SOAP study and ICON audit and performed a multivariable logistic regression analysis, with in-hospital death as the dependent variable. Covariates to be included in the final model were based on a univariate logistic regression analysis (*p* < 0.2) of demographic variables (age and sex), comorbid conditions, severity scores on admission to the ICU (SAPS II and SOFA scores), and severity of respiratory failure according to the PaO_2_/FiO_2_ ratio on the first day of mechanical ventilation. Colinearity between variables was ruled out before covariates were introduced in the model. Goodness of fit was tested using a Hosmer and Lemeshow test, and odds ratios (OR) with 95% confidence interval (CI) were computed. As driving pressure, Pplat, and PEEP are mathematically linked and were confirmed to be colinear (r^2^ > 0.6), we constructed separate logistic regression models for each parameter including the previously mentioned parameters. The multivariable models were adjusted for tidal volume > 8 ml/kg PBW, respiratory rate, the country of origin and the study period (ICON audit vs. SOAP study).

No statistical adjustments were used for multiple testing. All reported *p* values are two-sided and a *p* value < 0.05 was considered to indicate statistical significance.

## Results

### Temporal differences in the characteristics of patients with ARDS

The characteristics of the patients with ARDS included in the two cohorts are given in Table 1. The frequency of ARDS on admission to the ICU (5.1 vs. 5.0%, *p* = 0.866) and at any time during the ICU stay (10.4 vs. 10.7%, *p* = 0.793) was similar in the SOAP and ICON patients (Fig. 1). The diagnosis of ARDS was established at a median of 3 (IQ: 1–7) days after admission in the SOAP and 2 (IQ: 1–6) days in the ICON audit. Within 24 h of diagnosis, ARDS was mild in 244 (29.7%), moderate in 388 (47.3%), and severe in 189 (23.0%) patients.

**Table 1:** Characteristic of patients with acute respiratory distress syndrome (ARDS) on admission to the ICU

	SOAP study	ICON audit	*p* value
*N*	327	494	
Age, years, mean ± SD	59 ± 17	61 ± 16	0.124
Male, *n* (%)	194 (59.3)	321 (65)	0.079
Referring facility, *n* (%)			0.016
ER/ambulance	54 (18.5)	170 (34.4)	
Hospital floor	104 (35.6)	156 (31.6)	
OR/recovery room	77 (26.4)	72 (14.6)	
Other hospital	57 (19.5)	61 (12.3)	
Other	–	35 (7.1)	
Type of admission, *n* (%)			< 0.001
Medical admission	178 (54.4)	369 (74.7)	
Elective surgery	58 (17.7)	48 (9.7)	
Emergency surgery	91 (27.8)	77 (15.6)	
Severity scores, mean ± SD			
SAPS II score	47.7 ± 17.6	51.2 ± 17.1	0.002
SOFA score, total	7.9 ± 4.4	9.5 ± 4.1	< 0.001
Comorbidities, *n* (%)			
Non metastatic cancer	34 (10.4)	49 (9.9)	0.528
Diabetes, insulin dependent	18 (5.5)	43 (8.7)	0.087
Cirrhosis	11 (3.4)	26 (5.3)	0.199
Metastatic cancer	9 (2.8)	21 (4.3)	0.442
Hematologic cancer	19 (5.8)	25 (5.1)	0.641
Chemotherapy	6 (1.8)	25 (5.1)	0.023
HIV	0 (0)	8 (1.6)	0.025
Risk factors for ARDS, *n* (%)			
Shock, any cause	160 (48.9)	272 (55.1)	0.085
Polytrauma	28 (8.6)	35 (7.1)	0.436
Pancreatitis	12 (3.7)	17 (3.4)	0.862
Near drowning	2 (0.6)	0 (0)	0.082
Burns	2 (0.3)	0 (0)	0.219
PaO_2_/FiO_2_, mean ± SD	164 ± 90	172 ± 108	0.920

*ARDS* acute respiratory distress syndrome, *ER* emergency room, *HIV* human immunodeficiency virus, *OR* operating room, *SAPS* Simplified Acute Physiology Score, *SOFA* Sequential Organ Failure Assessment, *SD* standard deviation

Patients with ARDS in the later period (ICON audit) were more commonly admitted to the ICU for medical reasons than after surgical interventions (Table 1) and had slightly higher SAPS II and SOFA scores on admission to the ICU than those with ARDS included in the earlier study (SOAP) (Table 1).

#### Mechanical ventilation

**Fig. 2:**
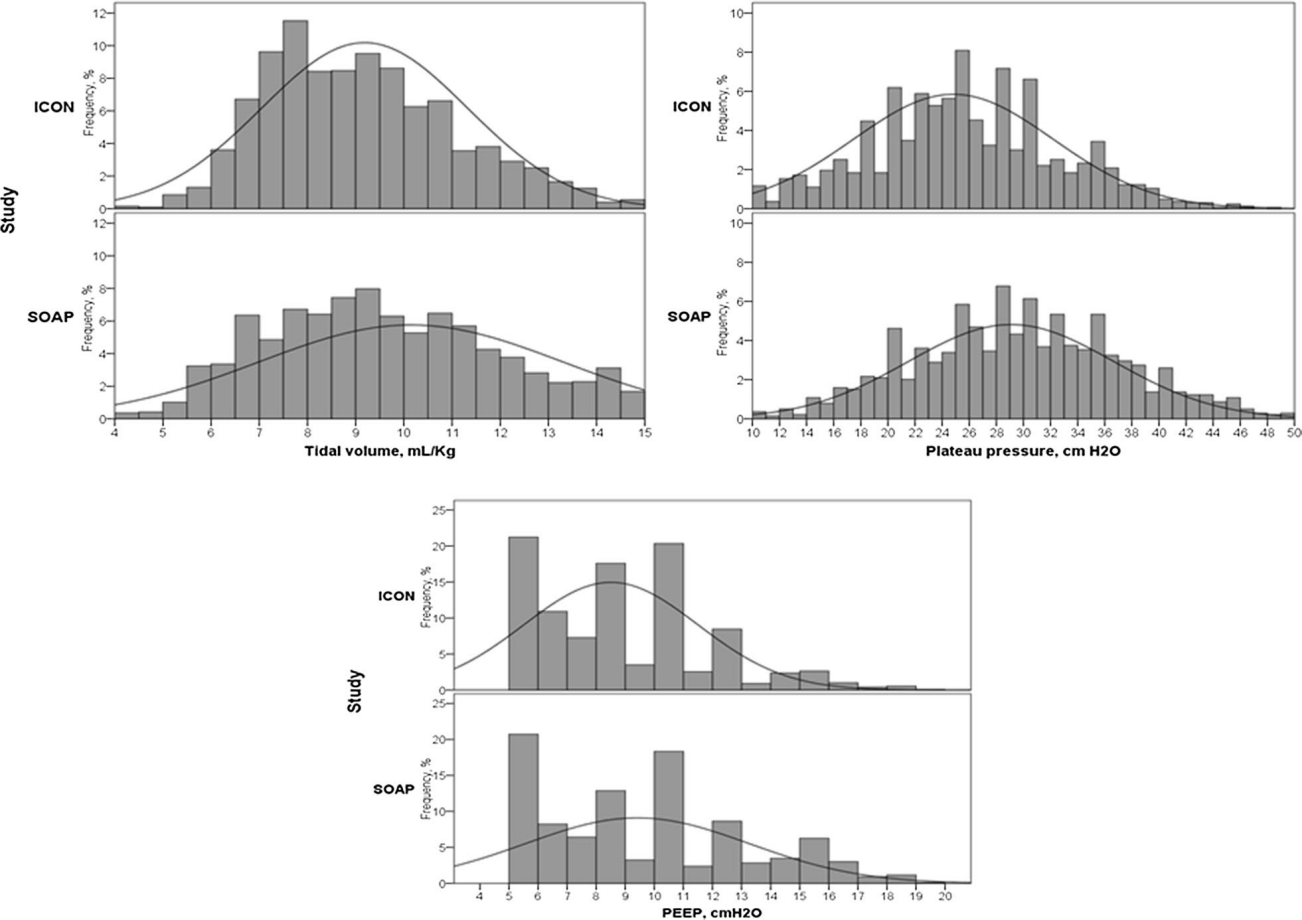
Histograms with normality curves representing the tidal volumes, plateau pressures, and positive end-expiratory pressures (PEEP) during mechanical ventilation in patients with acute respiratory distress syndrome (ARDS) in the SOAP and ICON studies

Ventilator settings in ARDS patients who required mechanical ventilation in the SOAP study and ICON audit are shown in Table 2. Respiratory rates were similar in the two cohorts. Tidal volumes were set at lower levels in the later (ICON) than in the earlier (SOAP) cohort (Fig. 2). Although the proportion of patients ventilated with protective tidal volumes (≤ 8 ml/kg) was higher in ICON than in SOAP (35.5% vs 18.0%, *p* < 0.001) and of patients ventilated with tidal volumes associated with VILI (i.e., > 10 ml/kg) was lower (117/465 [25.2%] vs 151/322 [46.9%], *p* < 0.001), after 10 years, more than 60% of patients with ARDS were still ventilated with tidal volumes greater than 8 ml/kg. PEEP was set at a slightly lower level in the ICON compared to the SOAP, and Pplat and driving pressure were also lower in the ICON audit than in the SOAP study (Table 2 and Fig. 2).

**Table 2:** Ventilatory parameters within 24 h of meeting ARDS criteria on mechanical ventilation in the two cohorts

	SOAP study		ICON audit		*p* value
	n*		n*		
Respiratory rate, mean ± SD		24 ± 21		23 ± 10	0.129
Tidal volume, ml/kg PBW, mean ± SD	322	10.3 ± 2.8	465	9.0 ± 2.1	< 0.001
< 6 ml/kg PBW, *n* (%)^†^		8 (2.5)		14 (3.0)	0.660
6–8 ml/kg PBW, *n* (%)^†^		50 (15.5)		151 (32.5)	< 0.001
8.1–10 ml/kg PBW, *n* (%)^†^		113 (35.1)		183 (39.4)	0.225
> 8 ml/kg PBW, *n* (%)^†^		264 (82.0)		300 (64.5)	< 0.001
> 10 ml/kg PBW, *n* (%)^†^		151 (46.9)		117 (25.2)	< 0.001
PEEP, cmH_2_O, mean ± SD	327	8.1 ± 3.4	494	7.6 ± 2.9	< 0.001
5–9 cmH_2_O, *n* (%)^†^		209 (63.9)		354 (71.7)	0.019
10–14 cmH_2_O, *n* (%)^†^		96 (29.4)		126 (25.5)	0.224
≥ 15 cmH_2_O, *n* (%)^†^		22 (6.7)		14 (2.8)	0.008
Plateau pressure, cmH_2_O, mean ± SD	284	28.0 ± 7.5	396	23.5 ± 7.1	< 0.001
> 29 cmH_2_O, *n* (%)^†^		116 (40.8)		80 (20.2)	< 0.001
Driving pressure, cmH_2_O, mean ± SD	284	19.9 ± 7.1	396	16.1 ± 6.1	< 0.001
> 14 cmH_2_O, *n* (%)^†^		219 (77.1)		236 (59.6)	< 0.001

*PBW* predicted body weight, *PEEP* positive end-expiratory pressure, *SD* standard deviation

*Number of patients with recorded values, excluding missing values

^†^Valid percentage after excluding missing values

#### Morbidity and mortality

The incidence of hepatic failure on admission to the ICU was higher and the incidence of renal failure lower in the ICON audit than in the SOAP study; the overall prevalence of hepatic, renal, and cardiovascular organ failure during the ICU stay was higher in the ICON audit than the SOAP study (Additional file 1: Table S2). ICU lengths of stay were similar in patients with ARDS in the two cohorts [median (IQ: 10 (5–21) vs. 9 (4–18) days, *p* = 0.257], whereas, hospital lengths of stay were longer in the SOAP study than ICON audit [median (IQ: 27 (11–55) vs. 16 (7–34) days, *p* < 0.001]. Hospital mortality rates in patients with mild, moderate and severe ARDS were not significantly different between the two studies (Additional file 1: Figure S1). Patients with severe ARDS within 24 h of diagnosis or at any time during the ICU stay had higher hospital mortality rates than those with mild and moderate ARDS. However, hospital mortality rates were similar in patients with mild and moderate ARDS during the ICU stay (Additional file 1: Figure S1).

### Predictors of worse outcome in patients with ARDS

In logistic regression analysis in all patients with ARDS from the two cohorts, with in-hospital death as the dependent variable, older age, greater SAPS II score, metastatic cancer, the presence of coagulation, renal and neurological system failures on admission to the ICU, and lower PaO_2_/FiO_2_ were independently associated with a greater risk of in-hospital death. Pplat > 29 cmH_2_O and driving pressure > 14 cmH_2_O on the first day of mechanical ventilation after establishing a diagnosis of ARDS, but not tidal volume > 8 ml/kg PBW or respiratory rate, were independently associated with a greater risk of death in these patients (Table 3).

**Table 3:** Logistic regression analysis with in-hospital death as the dependent variable in patients with ARDS

	Odds ratio (95% CI)	*p* value
Age (per year)	1.04 (1.03–1.04)	< 0.001
Male	0.81 (0.56–1.11)	0.235
SAPS II (per point)	1.04 (1.02–1.05)	< 0.001
Referring facility	–	–
ER/ambulance	R	NA
Hospital floor	0.89 (0.56–1.41)	0.618
OR/recovery room	0.72 (0.42–1.23)	0.618
Other hospital	0.77 (0.43–1.36)	0.367
Others	0.96 (0.39–2.34)	0.923
Comorbidities	–	–
No cancer	R	NA
Solid cancer, non-metastatic	0.88 (0.49–1.56)	0.653
Solid cancer, metastatic	2.90 (1.28–7.47)	0.027
Cirrhosis	1.70 (0.70–4.11)	0.244
Hematologic cancer	1.79 (0.75–4.23)	0.192
Steroids	1.52 (0.73–3.16)	0.263
Polytrauma	0.47 (0.21–1.06)	0.263
Organ failure on admission to the ICU	–	–
Coagulation	5.59 (2.74–11.41)	< 0.001
Hepatic	1.07 (0.58–1.97)	0.830
CNS	1.68 (1.14–2.48)	0.008
Renal	1.82 (1.20–2.76)	0.005
Cardiovascular	1.06 (0.73–1.53)	0.771
PaO_2_/FiO_2_ (per 20 mmHg)	0.99 (0.94–0.99)	< 0.001
Initial ventilatory settings	–	–
Respiratory rate	1.01 (0.99–1.02)	0.863
Tidal volume > 8.0 ml/kg PBW	0.68 (0.46–1.02)	0.157
PEEP, cmH_2_O^†^		
5–9	R	NA
10–14	1.1 (0.66–1.81)	0.72
≥ 15	2.61 (0.56–12.19)	0.224
Driving pressure > 14 cmH_2_O^†^	1.64 (1.1–2.46)	0.016
Plateau pressure > 29 cmH_2_O^†^	1.04 (1.01–1.07)	0.006

Excluding 96 patients with missing values and adjusted for country and study cohort (SOAP vs. ICON). Covariate inclusion in the final models was based on a univariate logistic regression analysis (*p* < 0.2) within the categories demographic variables (age and sex), comorbid conditions, severity of respiratory failure according to the PaO_2_/FiO_2_ ratio on the first day of mechanical ventilation, in addition to tidal volume and respiratory rate (Hosmer&Lemeshow goodness of fit Chi square: 5.49, *p* = 0.71; Nagelkerke’s R^2^ = 0.285). Patients who were excluded from the multivariable analysis due to missing variables (*n* = 96) had similar severity of respiratory failure as assessed by the PaO_2_/FiO_2_ ratio on the first day of mechanical ventilation and similar mortality rates compared to those who were included in the analysis

*CI* confidence interval, *CNS* central nervous system, *ER* emergency room, *ICU* intensive care unit, *OR* operating room, *PBW* predicted body weight; *PEEP* positive end-expiratory pressure, *SAPS* Simplified Acute Physiology Score

^†^Introduced alternately in different models due to colinearity. The displayed values refer to those considered in the model that included the PEEP as a covariate. Changes in the pressure parameters did not influence the significant p-values of the other covariates

## Discussion

The main findings of our study are: (1) the frequency of ARDS in European ICUs did not change significantly from 2002 to 2012 and morbidity and mortality rates were similarly high; (2) ventilation with lower tidal volumes and lower airway pressures (Pplat and driving pressure) increased over time; and (3) Pplat > 29 cmH_2_O and driving pressure > 14 cmH_2_O on the first day of mechanical ventilation but not tidal volume > 8 ml/kg PBW were independently associated with a higher risk of death in these patients.

In these two large European ICU cohorts [22, 23], performed 10 years apart, the frequency of ARDS at any time during the ICU stay remained relatively constant over time at just over 10%. Bellani et al. [8] reported that 10.4% of patients admitted to ICUs in 50 countries had ARDS during the ICU stay using the Berlin definitions [3]. Other studies [26–30] have reported a frequency of ARDS between 3 and 29%, varying according to the studied population and the definition used. Indeed, we previously reported that the frequency of ARDS was 12.6% from the SOAP study database [9] using the earlier European American Consensus criteria [2], which may overestimate the actual frequency of ARDS by including mild cases of respiratory dysfunction. Although we used the Berlin definitions to define ARDS [3], only patients requiring invasive mechanical ventilation were considered in our analysis due to the absence of precise data on non-invasive mechanical ventilation. Therefore, the overall frequency of ARDS in our study may have been slightly underestimated. Nonetheless, the same set of data were collected using similar protocols for the two cohorts [22, 23] and we only included data from patients admitted to ICUs in the same 24 countries.

Our data confirm the persistently high morbidity and mortality rates in patients with ARDS. Other studies have similarly reported mortality rates ranging from 40 to 60% in these patients [8, 31]. ARDS represents a major burden to the healthcare system, making it an important target for research into how best to manage these patients so as to improve outcomes. Despite increased adherence to a lung-protective strategy in mechanically ventilated patients with ARDS observed in the more recent ICON audit [23] compared to the earlier SOAP study [22], mortality rates did not seem to have improved. Other factors may, therefore, have played a role in determining the outcome in these patients. Indeed, we identified several factors, such as older age, greater SAPS II score, metastatic cancer, and the presence of coagulation, renal, and neurologic organ failures on admission to the ICU as being independently associated with a greater risk of in-hospital death. These factors, reflecting the severity of illness and the degree of organ dysfunction in these patients on admission to the ICU, have been reported in previous studies [9, 10].

Although the proportion of ARDS patients ventilated with low tidal volume (≤ 8 ml/kg PBW) and low Pplat (≤ 29 cmH_2_O) was higher in the later ICON audit than in the early SOAP study, a considerable proportion of ARDS patients in both studies were not mechanically ventilated using lung protective settings. One possible explanation for this gap between best evidence and practice is that ARDS may not have been adequately recognized by the clinicians in the ICUs contributing to the SOAP study and ICON audit. Indeed, a large observational study in ICU patients in 50 countries reported that only 34% of clinicians recognized ARDS at the time of actual fulfillment of ARDS criteria as assessed by a computer algorithm from raw data, suggesting that diagnosis of ARDS is frequently delayed [8]. These authors [8] also reported that ARDS was underdiagnosed, with only 60% of all patients with ARDS being recognized by the clinician. We may also assume that use of pressure-controlled mechanical ventilation may lead to inevitable fluctuations in tidal volume with possible transitory increases above the required limit of 8 ml/kg PBW. Calculation of tidal volume according to the actual weight rather than the PBW may also lead to erronously high tidal volume levels, especially in obese patients. Tidal volume > 8 ml/kg PBW on the first day of mechanical ventilation was not associated with the risk of death in patients with ARDS. This is perhaps not so surprising because the increased use of lower tidal volumes in the ICON audit decreased the median tidal volume in patients with ARDS included in the analysis (9 ml/kg PBW), which may have masked the potentially deleterious effects of high tidal volume observed in our previous analysis on the SOAP study database [9]. Airway pressures were also generally low, which may have outweighed the possible deleterious effects of high tidal volume. Low tidal volume remains, therefore, a main stay of ventilator management for these patients as supported by the best available evidence [2].

Pplat > 29 cmH_2_O on the first day of mechanical ventilation after establishing the diagnosis of ARDS was independently associated with the risk of death in these patients. Indeed, Pplat is an important determinant of lung overdistention [32] and a good indicator of lung stress [17], and higher levels are well correlated to the risk of barotrauma [33]. Therefore, limiting Pplat is a crucial component of lung-protective ventilation.

We also observed a potentially deleterious influence of driving pressure > 14 cmH_2_O on outcome. Amato et al. reported that driving pressure was the variable most strongly associated with mortality in a post-hoc analysis of data from nine randomized controlled trials of mechanically ventilated patients with ARDS [21]. Driving pressure > 14 cmH_2_O was also reported to be associated with an increased risk of hospital mortality in patients with moderate and severe ARDS [8]. Another study showed that driving pressure was associated with risk of death in hypoxemic patients regardless of the results of the chest radiograph or the presence of ARDS [34].

Our study has some limitations. First, this was a post hoc analysis and ARDS was not a primary or secondary outcome in either of our cohorts, and was not predefined in the SOAP or ICON surveys. The ventilation and physiologic parameters were recorded as the worst values during the day, so that baseline values at the onset of ARDS cannot be precisely determined. Nevertheless, the variables needed to define ARDS were collected prospectively by the two studies. In addition, although the Berlin definition of ARDS [3] addressed some limitations of the earlier AECC definition [2], poor reliability of some criteria may contribute to underrecognition by clinicians [35]. Second, the multivariable analysis is limited by the variables included and the effects of other non-reported variables cannot be excluded. However, we adjusted for a large number of factors that are known to influence outcomes in patients with ARDS. Third, a cause-effect relationship between the risk factors we reported and outcome cannot be ascertained due to the observational nature of the study. In this context, our data can be considered as hypothesis-generating to help guide future RCTs on the subject. Fourth, colinearity between the various airway pressure parameters due to a mathematical link between these parameters precluded their inclusion in the same multivariable model. Finally, ventilatory parameters were recorded at a fixed time point and may have changed during the day. Reporting of these parameters also did not follow specific instructions to standardize the timing of measurements within the respiratory cycle and the possible effect of spontaneous breathing cannot be fully excluded due to the observational nature of the study.

## Conclusion

The frequency of and outcome from ARDS remained unchanged between 2002 and 2012. The adoption of lower tidal volume in ARDS increased overtime and lower driving pressure and Pplat were observed in patients with ARDS included in the more recent ICON audit than in the earlier SOAP study. Pplat > 29 cmH_2_O and driving pressure > 14 cmH_2_O on the first day of mechanical ventilation, but not tidal volume > 8 ml/kg PBW, were independently associated with a higher risk of death.

## Supplementary Information

**Additional file 1**. Supplementary tables and figure.

## Data Availability

The datasets used and/or analyzed during the current study are available from the corresponding author on reasonable request.

## Abbreviations

ARDS: Acute respiratory distress syndrome
ECMO: Extracorporeal membrane oxygenation
ICON: Intensive Care over Nations
ICU: Intensive care unit
PEEP: Positive end-expiratory pressure
Pplat: Plateau pressure
SAPS II: Simplified Acute Physiology Score II
SOAP: Sepsis Occurrence in Acutely Ill Patients
SOFA: Sequential organ failure assessment
