# Supplementary material for: Temporal changes in the epidemiology, management, and outcome from acute respiratory distress syndrome in European intensive care units: a comparison of two large cohorts
Source: Crit Care. 2021 Feb 25;25:87. doi: 10.1186/s13054-020-03455-8 (PMC7906083; doi:10.1186/s13054-020-03455-8)

# Temporal Changes in the Epidemiology, Management, and Outcome from Acute Respiratory Distress Syndrome in European Intensive Care Units

Y Sakr et al

## Electronic Supplementary Material

# e-Appendix: List of SOAP and ICON investigators

## Alphabetical list of SOAP participating centers by country

*Austria:* University Hospital of Vienna (G. Delle Karth); LKH Steyr (V. Draxler); LKH-Deutschlandsberg (G. Filzwieser); Otto Wagner Spital of Vienna (W. Heindl); Krems of Donau (G. Kellner, T. Bauer); Barmherzige Bruede of Linz (K. Lenz); KH Floridsdorf of Vienna (E. Rossmann); University Hospital of Innsbruck (C. Wiedermann)
*Belgium:* CHU of Charleroi (P. Biston); Hôpitaux Iris Sud of Brussels (D. Chochrad); Clinique Europe Site St Michel of Brussels (V. Collin); C.H.U. of Liège (P. Damas); University Hospital Ghent (J. Decruyenaere, E. Hoste); CHU Brugmann of Brussels (J. Devriendt); Centre Hospitalier Jolimont-Lobbes of Haine St Paul (B. Espeel); CHR Citadelle of Liege (V. Fraipont); UCL Mont-Godinne of Yvoir (E. Installe); ACZA Campus Stuivenberg (M. Malbrain); OLV Ziekenhuis Aalst (G. Nollet); RHMS Ath-Baudour-Tournai (J.C. Preiser); AZ St Augustinus of Wilrijk (J. Raemaekers); CHU Saint-Pierre of Brussels (A. Roman); Cliniques du Sud-Luxembourg of Arlon (M. Simon); Academic Hospital Vrije Universiteit Brussels (H. Spapen); AZ Sint-Blasius of Dendermonde (W. Swinnen); Clinique Notre-Dame of Tournai (F. Vallot); Erasme University Hospital of Brussels (J.L. Vincent)
*Czech Republic:* University Hospital of Plzen (I. Chytra); U SV.Anny of Brno (L. Dadak); Klaudians of Mlada Boleslav (I. Herold); General Faculty Hospital of Prague (F. Polak); City Hospital of Ostrava (M. Sterba);
*Denmark :* Gentofte Hospital, University of Copenhagen (M. Bestle); Rigshospitalet of Copenhagen (K. Espersen); Amager Hospital of Copenhagen (H. Guldager); Rigshospitalet, University of Copenhagen (K-L. Welling)
*Finland:* Aland Central Hospital of Mariehamn (D. Nyman); Kuopio University Hospital (E. Ruokonen); Seinajoki Central Hospital (K. Saarinen)
*France:* Raymond Poincare of Garches (D. Annane); Institut Gustave Roussy of Villejuif (P. Catogni); Jacques Monod of Le Havre (G. Colas); CH Victor Jousselin of Dreux (F. Coulomb); Hôpital St Joseph & St Luc of Lyon (R. Dorne); Saint Joseph of Paris (M. Garrouste); Hôpital Pasteur of Nice (C. Isetta); CHU Brabois of Vandoeuvre Les Nancy (J. Larché); Saint Louis of Paris (J-R. LeGall); CHU de Grenoble (H. Lessire); CHU Pontchaillou of Rennes (Y. Malledant); Hôpital des Hauts Clos of Troyes (P. Mateu); CHU of Amiens (M. Ossart); Hôpital Lariboisière of Paris (D. Payen); CHD Félix Gyuon of Saint Denis La Reunion (P. Schlossmacher); Hôpital Bichat of Paris (J-F. Timsit); Hôpital Saint Andre of Bordeaux (S. Winnock); Hôpital Victor Dupouy of Argentueil (J-P. Sollet); CH Auch (L. Mallet); CHU Nancy-Brabois of Vandoeuvre (P. Maurer); CH William Morey of Chalon (JM. Sab); Victor Dupouy of Argenteuil (JP. Sollet)
*Germany:* University Hospital Heidelberg (G. Aykut); Friedrich Schiller University Jena (F. Brunkhorst); University Clinic Hamburg-Eppendorf (A. Nierhaus); University Hospital Mainz (M. Lauterbach); University Hospital Carl Gustav Carus of Dresden (M. Ragaller); Hans Sushemihl Krankenhaus of Emden (R. Gatz); Vivantes-Klinikum Neukoelln of Berlin (H. Gerlach); University Hospital RWTH Aachen (D. Henzler); Kreisklinik Langen-Seligenstadt (H-B Hopf**);** GKH Bonn (H. Hueneburg); Zentralklinik Bad Berka (W. Karzai); Neuwerk of Moenchengladbach (A. Keller); Philipps University of Marburg (U. Kuhlmann); University Hospital Regensburg (J. Langgartner); ZKH Links der Weser of Bremen (C. Manhold); University Hospital of Dresden (M. Ragaller); Universtiy of Wuerzburg (B. Reith); Hannover Medical School (T. Schuerholz); Universitätsklinikum Charité Campus Mitte of Berlin (C. Spies); Bethanien Hospital of Moers (R. Stögbauer); KhgmbH Schongau (J. Unterburger)
*Greece:* Thriassio Hospital of Athens (P-M. Clouva-Molyvdas); Sismanoglion General Hospital of Athens (G. Giokas); KAT General Hospital of Athens (E. Ioannidou); G. Papanikolaou General Hospital of Thessaloniki (A. Lahana); Agios Demetrios of Thessaloniki (A. Liolios); Onassis Cardiac Surgery Center of Athens (K. Marathias); University Hospital of Ioannina (G. Nakos); Tzanio Hospital of Athens (A. Tasiou); Athens General Hospital Gennimatas (H. Tsangaris)
*Hungary:* Peterfy Hospital of Budapest (P. Tamasi)
*Ireland:* Mater Hospital of Dublin (B. Marsh); Beaumont Hospital of Dublin (M. Power)
*Israel:* Hadassah Hebrew University Medical Center (C. Sprung)
*Italy:* Azienda Ospedaliera Senese o Siena (B. Biagioli); S. Martino of Genova (F. Bobbio Pallavicini); Azienda Ospedaliera S. Gerardo dei Tintori of Monza (A. Pesenti); Osp Regionale of Saronno (C. Capra); Ospedale Maggiore - University A. Avogadro of Novara (F. Della Corte); Osp. Molinette of Torino (P. P. Donadio); A.O. Umberto I Ancona, Rianimazione Clinica (A. Donati); Azienda Ospedaliera Universitaria Policlinico of Palermo (A. Giarratano); San Giovanni Di Dio of Florence (T. Giorgio); H San Raffaele IRCCS of Milano (D. Giudici); Ospedale Di Busto Arsizio (S. Greco); Civile Di Massa (A. Guadagnucci); San Paolo

of Milano (G. Lapichino); S.Giovanni Bosco Torino (S. Livigni); Osp. San Giovanni of Sesto (G. Moise); S Camillo of Roma (G. Nardi); Vittorio Emanuele of Catania (E. Panascia); Hospital of Piacenza (M. Pizzamiglio); Universita di Torino-Ospedale S. Giovanni Battista (V. M. Ranieri); Policlinico Le Scotte of Siena (R. Rosi); Ospedale Maggiore Policlinico IRCCS of Milano (A. Sicignano); A. Uboldo of Cernusco Sul Naviglio (M. Solca); P.O. Civile Carrara of Massa (G. Vignali); San Giovanni of Roma (I. Volpe Rinonapoli)
*Netherlands:* Boven IJ Ziekenhuis of Amsterdam (M. Barnas); UMC St Radboud of Nijmegen (E.E. De Bel); Academic Medical Center of Amsterdam (A-C. De Pont); VUMC of Amsterdam (J. Groeneveld); Groningen University Hospital (M Nijsten); Waterlandziekenhuis of Purmerend (L Sie); OLVG of Amsterdam (D. F. Zandstra)
*Norway:* Sentralsjukehuset i Rogaland of Stavanger (S. Harboe); Sykehuset Østfold of Fredrikstad (S. Lindén); Aker University Hospital of Oslo (R. Z. Lovstad); Ulleval University Hospitalof Oslo (H. Moen); Akershus University Hospital of Nordbyhagen (N. Smith-Erichsen)
*Poland:* Paediatric University Hospital of Lodz (A. Piotrowski); Central Clinic Hospital SLAM of Katowice (E. Karpel)
*Portugal :* Garcia de Orta of Almada (E. Almeida); Hospital de St. António dos Capuchos of Lisboa (R. Moreno); Hospital de Santa Maria of Lisboa (A. Pais-De-Lacerda); Hospital S.Joao of Porto (J. A. Paiva); Fernado Fonseca of Masama (I. Serra); São Teotonio Viseu (A. Pimentel)
*Romania:* Inst of Cardiovascular Diseases of Bucharest (D. Filipescu)
*Serbia:* Military Medical Academy of Belgrade (K. Jovanovic)
*Slovakia:* SUSCH of Bratislava (P. Malik)
*Slovenia:* General Hospital of Novo Mesto (K. Lucka); General Hospital of Celje (G. Voga)
*Spain:* Hospital Universitario Rio Hortega of Valladolid (C. Aldecoa Alvarez-Santullano); Sabadell Hospital (A. Artigas); Hospital Clinic of Barcelona (E. Zavala, A. Escorsell, J. Nicolas); Virgen del Camino of Pamplona (J. J. Izura Cea); Virgen de la Salud of Toledo (L. Marina); 12 de Octubre of Madrid (J. Montejo); Gregorio Maranon of Madrid (E. Palencia); General Universitario de Elche (F. Santos); Puerta del Mar of Cadiz (R. Sierra-Camerino); Fundación Jiménez Díaz of Madrid (F. Sipmann); Hospital Clinic of Barcelona (E. Zavala)
*Sweden:* Central Hospital of Kristianstad (K. Brodersen); Stockholm Soder Hospital (J. Haggqvist); Sunderby Hospital of Luleå (D. Hermansson); Huddinge University Hospital of Stockholm (H. Hjelmqvist)
*Switzerland:* Kantonsspital Luzern (K. Heer); Hirslanden Klinik Beau-Site of Bern (G. Loderer); University Hospital of Zurich (M. Maggiorini); Hôpital de la ville of La Chaux-de-Fonds (H. Zender)
*United Kingdom:* Edinburgh Western General Hospital (P. Andrews); Peterborough Hospitals NHS Trust of Peterborough (B. Appadu); University Hospital Lewisham, London (C. Barrera Groba); Bristol Royal Infirmary (J. Bewley); Queen Elizabeth Hospital Kings Lynn (K. Burchett); Milton Keynes General (P. Chambers); Homerton University Hospital of London (J. Coakley); Charing Cross Hospital of London (D. Doberenz); North Staffordshire Hospital of Stoke On Trent (N. Eastwood); Antrim Area Hospital (A. Ferguson); Royal Berkshire Hospital of Reading (J. Fielden); The James Cook University Hospital of Middlesbrough (J. Gedney); Addenbrookes of Cambridge (K. Gunning); Rotherham DGH (D. Harling); St.Helier of Carshalton (S. Jankowski); Southport & Formby (D. Jayson); Freeman of Newcastle Upon Tyne (A. Kilner); University Hospital of North Tees at Stockton on Tees (V. Krishna-Kumar); St. Thomas Hospital of London (K. Lei); Royal Infirmary of Edinburgh (S. Mackenzie); Derriford of Plymouth (P. Macnaughton); Royal Liverpool University Hospital (G. Marx); Stirling Royal Infirmary (C. McCulloch); University Hospital of Wales, Cardiff (P. Morgan); St George's Hospital of London (A. Rhodes); Gloucestershire Royal Hospital (C. Roberts); St Peters of Chertsey (M. Russell); James Paget Hospital of Great Yarmouth (D. Tupper-Carey, M. Wright); Kettering General Hospital (L. Twohey); Burnley DGH (J. Watts); Northampton General Hospital (R. Webster); Dumfries Royal Infirmary (D. Williams)

## Alphabetical list of ICON participating centers by country (for the same countries as were included in SOAP)

*Austria*: Akh Wien (P Urbanek); Allgemeines Und Orthopädisches Landeskrankenhaus Stolzalpe (J Schlieber); Barmherzige Schwestern Linz (J Reisinger); General Hospital Braunau (J Auer); Krankenhaus D. Barmherzigen Schwestern Ried I.I. (A Hartjes); Krankenhaus Floridsdorf (A Lerche); LK Gmünd-Waidhofen/Thaya-Zwettl, Standort Zwettl (T Janous); LKH Hörgas-Enzenbach (E Kink); LKH West (W Krahulec); University Hospital (K Smolle)

*Belgium*: AZ Groeninge Kortrijk (M Van Der Schueren); AZ Jan Palfijn Gent (P Thibo); AZ Turnhout (M Vanhoof); Bracops Anderlecht (I Ahmet); Centre Hospitalier Mouscron (G Philippe); CH Peltzer La Tourelle (P Dufaye); Chirec Edith Cavell (O Jacobs); CHR Citadelle (V Fraipont); CHU Charleroi (P Biston); Chu Mont-Godinne (A Dive); CHU Tivoli (Y Bouckaert); Chwapi (E Gilbert); Clinique Saint-Pierre Ottignies (B Gressens); Clinique-Maternité Sainte Elisabeth (E Pinck); Cliniques De L'Europe - St-Michel (V Collin); Erasme University Hospital (JL Vincent); Ghent University Hospital (J De Waele); Moliere Hospital (R Rimachi); Notre Dame (D Gusu); Onze Lieve Vrouw Ziekenhuis, Aalst (K De Decker); Ixelles Hospital (K Mandianga); Sint-Augustinus (L Heytens); St Luc University Hospital (UCL) (X Wittebole); UZ Brussel (S Herbert); Vivalia Site De Libramont (V Olivier); VZW Gezondheidszorg Oostkust Knokke-Heist (W Vandenheede); ZNA Middelheim (P Rogiers)
*Czech Republic*: Centre of Cardiovascular and Transplant Surgery (P Pavlik); Charles University Hospital (J Manak); IKEM, Prague (E Kieslichova); KNTB Zlín A.S. (R Turek); Krajska Nemocnice Liberec (M Fischer); Masarykova Nemocnice V Usti Nad Labem (R Valkova); St. Anne's University Hospital Brno (L Dadak); University Hospital Haradec Králové (P Dostal); University Hospital Brno (J Malaska); University Hospital Olomouc (R Hajek); University Hospital Plzen (A Židková); Charles University Hospital Plzen (P Lavicka)
*Denmark:* Herning Hospital (P Kolodzeike); Hjoerring Hospital (M Kruse); Vejle Hospital (T Andersen)
*Finland*: Helsinki University Central Hospital (V Harjola); Seinäjoki Central Hospital (K Saarinen)
*France*: Aix Marseille Univ, Hôpital Nord (M Leone); Calmette Hospital, Lille (A Durocher); Centre Hospitalier de Dunkerque (S Moulront); Centre Hospitalier Lyon Sud (A Lepape); Centre Hospitalo-Universitaire Nancy-Brabois (M Losser); CH Saint Philibert, Ghicl, Lille (P Cabaret); CHR De Dax (E Kalaitzis); CHU Amiens (E Zogheib); CHU Dijon (P Charve); CHU Dupuytren (B Francois); CHU Nîmes (JY Lefrant); Centre Hospitalier De Troyes (B Beilouny); Groupe Hospitalier Est Francilien-Centre Hospitalier De Meaux (X Forceville); Groupe Hospitalier Paris Saint Joseph (B Misset); Hôpital Antoine Béclère (F Jacobs); Hôpital Edouard Herriot (F Bernard); Hôpital Lariboisère, APHP, Paris France (D Payen); Hôpital Maison Blanche, Reims (A Wynckel); Hopitaux Universitaires de Strasbourg (V Castelain); Hospices Civils de Lyon (A Faure); CHU-Grenoble (P Lavagne); CHU-Nantes (L Thierry); Réanimation, CHU Lille (M Moussa); University Hospital Ambroise Paré (A Vieillard-Baron); University Hospital Grenoble (M Durand); University Hospital of Marseille (M Gainnier); University of Nice (C Ichai)
*Germany*: Alexianer Krefeld Gmbh (S Arens); Charite Hochschulmedizin Berlin (C Hoffmann); Charite-University-Hospital, Berlin (M Kaffarnik); Diakoniekrankenhaus Henriettenstiftung Gmbh (C Scharnofske); Elisabeth-Krankenhaus Essen (I Voigt); Harlaching Hospital, Munich Municipal Hospital Group (C Peckelsen); Helios St. Johannes Klinik (M Weber); Hospital St. Georg Leipzig (J Gille); Klinik Hennigsdorf Der Oberhavel Kliniken Gmbh (A Lange); Klinik Tettnang (G Schoser); Klinikum "St. Georg" Leipzig (A Sablotzki); Klinikum Augsburg (U Jaschinski); Klinikum Augsburg (A Bluethgen); Klinikum Bremen-Mitte (F Vogel); Klinikum Bremen-Ost (A Tscheu); Klinikum Heidenheim (T Fuchs); Klinikum Links Der Weser Gmbh (M Wattenberg); Klinikum Luedenscheid (T Helmes); Krankenhaus Neuwerk (S Scieszka); Marienkrankenhaus Schwerte (M Heintz); Medical Centre Cologne Merheim (S Sakka); Schwarzwald-Baar Klinikum Villingen-Schwenningen (J Kohler); St. Elisabeth Krankenhaus Köln-Hohenlind (F Fiedler); St. Martinus Hospital Olpe (M Danz); Uniklinikum Jena (Y Sakr); Universitätsklinikum Tübingen (R Riessen); Universitätsmedizin Mainz (T Kerz); University Hospital Aachen, CPACC (A Kersten); University Hospital Aachen, DMIII (F Tacke); University Hospital Aachen, OIC (G Marx); University Hospital Muenster (T Volkert); University Medical Centre Freiburg (A Schmutz); University Medical Centre Hamburg-Eppendorf (A Nierhaus); University Medical Centre Hamburg-Eppendorf (S Kluge); University Medicine Greifswald (P Abel); University of Duisburg-Essen (R Janosi); University of Freiburg (S Utzolino); University clinic Ulm (H Bracht); Vivantes Klinikum Neukoelln (S Toussaint)
*Greece*: Ahepa University Hospital (M Giannakou Peftoulidou); Athens University (P Myrianthefs); Athens University Medical School (A Armaganidis); Evangelismos Hospital (C Routsi); General Hospital of Chania, Crete (A Xini); Hippokration General Hospital, Thessaloniki (E Mouloudi); General hospital of Velos (I Kokoris); Lamia General Hospital (G Kyriazopoulos); Naval and Veterans Hospital (S Vlachos); Papanikolaou General Hospital (A Lavrentieva); University Hospital Alexandroupolis (P Partala); University of Ioannina (G Nakos)

*Hungary*: Dr. Kenessey Albert Hospital (L Medve); Fejér County St George Teaching Hospital (A Sarkany); Flor Ferenc County Hospital (I Kremer); Jávorszky Ödön Hospital (Z Marjanek); Peterfy Hospital Budapest (P Tamasi)
*Ireland*: Cork University Hospital (J Barry); Mercy University Hospital (R O'Leary); Mid Western Regional Hospital Complex (C Motherway); Midland Regional Hospital Mullingar, Co Westmeath (M Faheem); St. Vincent's University Hospital (E Dunne); Tallaght Hospital (M Donnelly); University Hospital Galway (T Konrad)
*Israel*: Rabin Medical Centre (J Cohen); Sourasky Tel Aviv Medical Centre (O Sold)
*Italy*: Anesthesiology and Intensive Care (E Bonora); AO Ospedale Niguarda Ca' Granda (C Achilli); Azienda Ospedaliera Di Padova (S Rossi); Azienda Ospedaliero Universitaria Policlinico Vittorio Emanuele (G Castiglione); Careggi Teaching Hospital (A Peris); Clinicized Hospital Ss Annunziata - Chieti (D Albanese); Fondazione Irccs Ca' Granda Ospedale Maggiore Policlinico, Milano; University of Milan (N Stocchetti); H San Gerardo - Monza (G Citerio); Icu "Ceccarini" Hospital Riccione (L Mozzoni); Irccs Centro Cardiologico Monzino (E Sisillo); Irccs Centro Di Riferimento Oncologico Della Basilicata (P De Negri); Irccs Fondazione Ca' Granda - Ospedale Maggiore Policlinico (M Savioli); Ospedale Belcolle Viterbo (P Vecchiarelli); Ospedale Civile Maggiore - A.O.U.I Verona (F Puflea); Ospedale Civile Maggiore - A.O.U.I Verona (V Stankovic); Ospedale Di Circolo E Fondazione Macchi - Varese (G Minoja); Ospedale Di Trento - Azienda Provinciale Per I Servizi Sanitari Della Provincia Autonoma Di Trento (S Montibeller); Ospedale Orlandi (P Calligaro); Ospedale Regionale U.Parini-Aosta (R Sorrentino); Ospedale San Donato Arezzo (M Feri); Ospedale San Raffaele (M Zambon); Policlinico G.B. Rossi - A.O.U.I Verona (E Colombaroli); Policlinico University of Palermo (A Giarratano); Santa Maria Degli Angeli Hospital (T Pellis); Saronno Hospital (C Capra); Università Cattolica Del Sacro Cuore (M Antonelli); University Catania, Italy (A Gullo); University of Florence, Florence (C Chelazzi); University of Foggia (A De Capraris); University of Milano-Bicocca, San Gerardo Hospital (N Patroniti); University of Modena (M Girardis); University of Siena (F Franchi); University of Trieste (G Berlot)
*Netherlands*: Albert Schweitzer Hospital (H Ponssen); Antoni Van Leeuwenhoek Ziekenhuis (J Ten Cate); Atrium Medisch Centrum Parkstad (L Bormans); Bovenij Hospital (S Husada); Catharina Hospital Eindhoven (M Buise); Erasmus University Medical Centre (B Van Der Hoven); Martiniziekenhuis Groningen (A Reidinga); Medical Centre Leeuwarden (M Kuiper); Radboud University Nijmegen Medical Centre (P Pickkers); Slotervaart Ziekenhuis Amsterdam (G Kluge); Spaarne Ziekenhuis (S Den Boer); University Medical Centre Utrecht (J Kesecioglu); Ziekenhuis Rijnstate (H Van Leeuwen)
*Norway*: Haukeland University Hospital (H Flaatten); St Olavs Hospital, Trondheim University Hospital (S Mo)
*Poland*: Csk Mswia (J Kolbusz); Medical University (A Kübler); Medical University Of Wroclaw (B Mielczarek); Medical University Warsaw (M Mikaszewska-Sokolewicz); Pomeranian Medical University (K Kotfis); Regional Hospital in Poznan (B Tamowicz); Szpital Powiatowy W Ostrowi Mazowieckiej (W Sulkowski); University Hospital, Poznam (P Smuszkiewicz); Wojewódzki Szpital Zakazny (A Pihowicz); Wojewódzkie Centrum Medyczne (E Trejnowska)
*Portugal*: Centro Hospitalar Cova Da Beira (V Branco); Centro Hospitalar Do Porto (F Rua); Centro Hospitalar Do Tâmega E Sousa (E Lafuente); Centro Hospitalar Gaia/Espinho, Epe (M Sousa); Centro Hospitalar Médio Tejo (N Catorze); Centro Hospitalar Tondela-Viseu (M Barros); Faro Hospital (L Pereira); Hospital Curry Cabral (A Vintém De Oliveira); Hospital Da Luz (J Gomes); Hospital De Egas Moniz - Chlo (I Gaspar); Hospital De Santo António, Centro Hospitalar Do Porto (M Pereira); Hospital Divino Espírito Santo, Epe (M Cymbron); Hospital Espirito Santo - Évora Epe (A Dias); Hospital Garcia Orta (E Almeida); Hospital Geral Centro Hospitalar E Universitario Coimbra (S Beirao); Hospital Prof. Doutor Fernando Fonseca Epe (I Serra); Hospital São Bernardo (R Ribeiro); Hospital Sao Francisco Xavier, Chlo (P Povoa); Instituto Portugues De Oncologia Francisco Gentil, Porto (F Faria); Santa Maria Hospital (Z Costa-E-Silva); Serviço De Saúde Da Região Autonóma Da Madeira (J Nóbrega); UCIP (F Fernandes); ULS - Castelo Branco (J Gabriel)
*Romania*: Emergency County Hospital Cluj (N Hagau); Emergency Institute for Cardiovascular Diseases (D Filipescu); Fundeni Clinical Institute (G Droc); Galati Hospital (M Lupu); Inbi "Prof. Dr. Matei Bals" (A Nica); Institute of Pulmonology Marius Nasta (R Stoica); Institutul Clinic Fundeni (D Tomescu); Sfantul Pantelimon Hospital (D Constantinescu); Spitalul Cf 2 Bucuresti (G Valcoreanu Zbaganu); “Luliu

Hatieganu" University of Medicine and Pharmacy, Teaching Hospital of Infectious Diseases, Cluj-Napoca (A Slavcovici)
*Serbia*: Clinic for Cardiac Surgery, Clinical Centre of Serbia (L Soskic); Clinic for Digestive Surgery, Clinical Centre Serbia (I Palibrk); Clinic for Vascular Surgery, Clinical Centre Nis (R Jankovic); Clinical Centre of Serbia (B Jovanovic); Clinical Centre of Serbia (M Pandurovic); Emergency Centre, Clinical Centre of Belgrade (V Bumbasirevic); General University Hospital (B Uljarevic); Military Medical Academy (M Surbatovic); Urology Hospital (N Ladjevic)
*Slovakia*: District Hospital (G Slobodianiuk); Faculty Hospital (V Sobona); University Hospital Bratislava-Hospital Ruzinov ICU (A Cikova); University Hospital Ruzinov Bratislava (A Gebhardtova)
*Slovenia*: General Hospital Celje (G Voga); General Hospital Izola (E Rupnik); General Hospital Novo Mesto (L Kosec); Oncological Institute (M Kerin Povšic); Ukc Maribor (I Osojnik); University Clinic of Respiratory and Allergic Diseases (V Tomic); University Clinical Centre Maribor (A Sinkovic)
*Spain*: CH Salamanca (J González); Clinic Hospital (E Zavala); Complejo Hospitalario De Jaén (J Pérez Valenzuela); Complejo Hospitalario De Toledo (L Marina); Complexo Hospitalario Universitario De Ourense (P Vidal-Cortés); Complexo Hospitalario Universitario De Vigo (P Posada); Corporación Sanitaria Parc Tauli (A Ignacio Martin-Loeches); Cruz Roja Hospital (N Muñoz Guillén); H Vall Hebron (M Palomar); HGGC Dr Negrín (J Sole-Violan); Hospital Clinic (A Torres); Hospital Clinico San Carlos (M Gonzalez Gallego); Hospital Clínico Universitario De Valencia (G Aguilar); Hospital Clínico Universitario Lozano Blesa (R Montoiro Allué); Hospital Clinico Valencia (M Argüeso); Hospital De La Ribera (M Parejo); Hospital De Sagunto (M Palomo Navarro); Hospital De San Juan De Alicante (A Jose); Hospital De Torrejon De Ardoz (N Nin); Hospital Del Mar (F Alvarez Lerma); Hospital Del Tajo (O Martinez); Hospital General Universitario De Elche (E Tenza Lozano); Hospital General Universitario Gregorio Marañon (S Arenal López); Hospital General Universitario Gregorio Marañon (M Perez Granda); Hospital General Universitario Santa Lucía (S Moreno); Hospital Germans Trias I Pujol (C Llubia); Hospital Infanta Margarita (C De La Fuente Martos); Hospital Infanta Sofia (P Gonzalez-Arenas); Hospital J.M. Morales Meseguer (N Llamas Fernández); Hospital J.M. Morales Meseguer (B Gil Rueda ); Hospital Marina Salu. Denia. Alicante. (I Estruch Pons); Hospital Nuestra Señora Del Prado, Talavera De La Reina, Toledo. España (N Cruza); Hospital San Juan De Dios Aljarafe (F Maroto); Hospital Sas of Jerez (A Estella); Hospital Son Llatzer (A Ferrer); Hospital Universitario Central De Asturias (L Iglesias Fraile); Hospital Universitario Central De Asturias (B Quindos); Hospital Universitario De Alava, Santiago (A Quintano); Hospital Universitario De Basurto, Bilbao (M Tebar); Hospital Universitario de Getafe (P Cardinal); Hospital Universitario De La Princesa (A Reyes); Hospital Universitario de Tarragona Joan Xxiii (A Rodríguez); Hospital Universitario Del Henares (A Abella); Hospital Universitario Fundación Alcorcón (S García Del Valle); Hospital Universitario La Paz (S Yus); Hospital Universitario La Paz (E Maseda); Hospital Universitario Rio Hortega (J Berezo); Hospital Universitario San Cecilio (Granada) (A Tejero Pedregosa); Hospital Virgen Del Camino (C Laplaza); Mutua Terrassa University Hospital (R Ferrer); Rão Hortega University Hospital (J Rico-Feijoo); Servicio Andaluz De Salud. Spain. (M Rodríguez); University Opf Navarra (P Monedero)
*Sweden*: Karolinska University Hospital And Karolinska Institute (K Eriksson); Sunderby Hospital, Luleå (D Lind)
*Switzerland*: Hôpital Intercantonal De La Broye (D Chabanel); Hôpital Neuchâtelois - La Chaux-De-Fonds (H Zender); Lindenhofspital (K Heer); Regionalspital Surselva Ilanz (Gr) Schweiz (B Frankenberger); University Hospital Bern (S Jakob); Zentrum Für Intensivmedizin (A Haller)
*United Kingdom*: Alexandra Hospital Redditch (S Mathew); Blackpool Teaching Hospitals (R Downes); Brighton And Sussex University Hospitals (C Barrera Groba); Cambridge University Hospitals NHS Foundation Trust (A Johnston); Charing Cross Hospital (R Meacher); Chelsea & Westminster Hospital (R Keays); Christie Foundation Trust (P Haji-Michael); County Hospital, Lincoln (C Tyler); Craigavon Area Hospital (A Ferguson); Cumberland Infirmary (S Jones); Darent Valley Hospital (D Tyl); Dorset County Hospital (A Ball); Ealing Hospital NHS Trust (J Vogel); Glasgow Royal Infirmary (M Booth); Gloucester Royal Hospital (P Downie); The Great Western Hospital, Swindon (M Watters); Imperial College Healthcare NHS Trust (S Brett); Ipswich Hospital Nhs Trust (M Garfield); James Paget University Hospital NHS Foundation Trust (L Everett); King's College Hospital (S Heenen); King's Mill Hospital (S Dhir); Leeds Teaching Hospitals NHS Trust (Z Beardow); Lewisham Healthcare NHS Trust (M Mostert); Luton and

Dunstable Hospital NHS Trust (S Brosnan); Medway Maritime Hospital (N Pinto); Musgrove Park Hospital (S Harris); Nevill Hall Hospital (A Summors); Pilgrim Hospital (N Andrew); Pinderfields Hospital, Mid Yorkshire NHS Trust (A Rose); Plymouth Hospitals Nhs Trust (R Appelboam); Princess Royal Hospital Telford (O Davies); Royal Bournemouth Hospital (E Vickers); Royal Free Hampstead NHS Foundation Trust (B Agarwal); Royal Glamorgan Hospital (T Szakmany); Royal Hampshire County Hospital (S Wimbush); Royal Liverpool University Hospital (I Welters); Royal London Hospital, Barts Health NHS Trust (R Pearse); Royal Shrewsbury Hospital (R Hollands); Royal Surrey County Hospital (J Kirk-Bayley); St Georges Healthcare (N Fletcher); Surrey & Sussex Healthcare Trust (B Bray); University College Hospital (D Brealey)

**Table S1** Countries contributing to both the SOAP study and the ICON audit with the corresponding numbers of patients and patients with ARDS

| | SOAP study | | ICON audit | |
|---|---|---|---|---|
| | All, n | ARDS, n (%) | All, n | ARDS, n (%) |
| Austria | 68 | 5 (7.4) | 61 | 0 (0) |
| Belgium | 703 | 31 (4.4) | 481 | 48 (10) |
| Czech Republic | 45 | 3 (6.7) | 106 | 12 (11.2) |
| Denmark | 29 | 8 (27.6) | 49 | 3 (6.1) |
| Finland | 51 | 3 (5.9) | 32 | 3 (9.4) |
| France | 332 | 35 (10.5) | 327 | 34 (10.4) |
| Germany | 329 | 21 (6.4) | 659 | 43 (6.5) |
| Greece | 109 | 23 (21.1) | 45 | 14 (31.1) |
| Hungary | 8 | 0 (0) | 55 | 4 (7.3) |
| Ireland | 33 | 11 (33.3) | 75 | 15 (20) |
| Israel | 13 | 2 (15.4) | 23 | 6 (26.1) |
| Italy | 237 | 25 (10.5) | 299 | 46 (15.4) |
| Netherlands | 144 | 9 (6.3) | 219 | 15 (6.8) |
| Norway | 61 | 6 (9.8) | 29 | 7 (24.1) |
| Poland | 13 | 0 (0) | 54 | 16 (29.6) |
| Portugal | 69 | 13 (18.8) | 188 | 32 (17) |
| Romania | 44 | 4 (9.1) | 189 | 13 (6.9) |
| Serbia | 2 | 2 (100) | 126 | 14 (11.1) |
| Slovakia | 3 | 0 (0) | 17 | 3 (17.6) |
| Slovenia | 46 | 1 (2.2) | 78 | 6 (7.7) |
| Spain | 202 | 24 (11.9) | 574 | 45 (7.8) |
| Sweden | 68 | 1 (1.5) | 37 | 3 (8.1) |
| Switzerland | 114 | 3 (2.6) | 151 | 10 (6.6) |
| United Kingdom | 424 | 97 (22.9) | 727 | 102 (14) |
| All patients | 3147 | 327 ( 10.4) | 4601 | 494 ( 10.7) |

**Table S2** Non-respiratory organ failure as assessed by the sequential organ failure assessment (SOFA) score and organ support

| | SOAP STUDY | ICON AUDIT | P value |
|---|---|---|---|
| N | 327 | 494 | |
| On admission, n (%) | | | |
| Coagulation | 36 (11) | 47 (9.5) | 0.487 |
| Hepatic | 19 (5.8) | 67 (13.6) | <0.001 |
| CNS | 107 (32.7) | 172 (34.8) | 0.548 |
| Renal | 91 (27.8) | 107 (21.7) | 0.043 |
| Cardiovascular | 160 (48.9) | 272 (55.1) | 0.089 |
| At any time, n (%) | | | |
| Coagulation | 87 (26.6) | 106 (21.5) | 0.089 |
| Hepatic | 43 (13.1) | 161 (32.6) | <0.001 |
| CNS | 149 (45.6) | 257 (52) | 0.071 |
| Renal | 168 (51.4) | 296 (59.9) | 0.016 |
| Cardiovascular | 238 (72.8) | 399 (80.8) | 0.007 |
| Organ support on admission, n (%) | | | |
| Renal replacement therapy | 23 (7) | 40 (8.1) | 0.575 |
| Mechanical ventilation | 290 (88.7) | 411 (83.2) | 0.092 |
| Organ support, any time, n (%) | | | |
| Renal replacement therapy | 79 (24.2) | 140 (28.3) | 0.185 |
| Mechanical ventilation | 327 (100) | 494 (100) | 1.00 |
| SOFA score (non-respiratory), mean ± SD | | | |
| SOFA score on ICU admission | 8.6±3.9 | 6.8±3.7 | <0.001 |
| SOFAmean | 5.6±3.6 | 6.4±3.6 | 0.001 |
| SOFAmax | 8.6±3.9 | 9.6±3.9 | <0.001 |

CNS: central nervous system, ICU: intensive care unit, SOFAmean: mean SOFA score during the ICU stay, SOFAmax: maximum SOFA score in the ICU

**Figure S1**. Bar chart representing hospital mortality rates according to the study cohort and the severity of ARDS, a) within 24 hours of diagnosis and b) the worst category during the ICU stay. N= number of patients in each group

a)

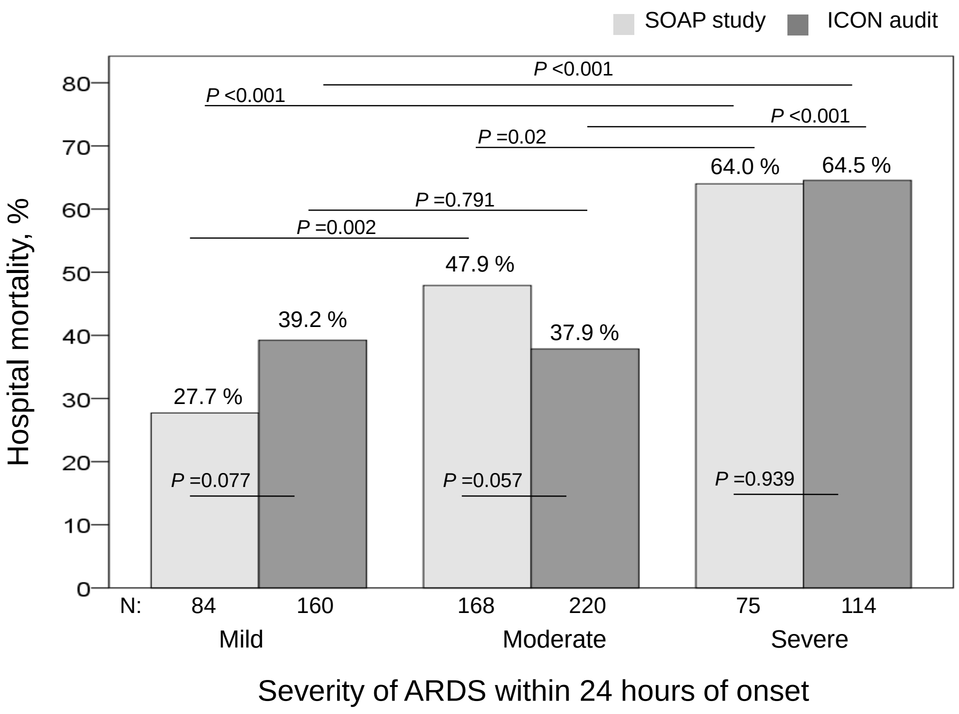


b)

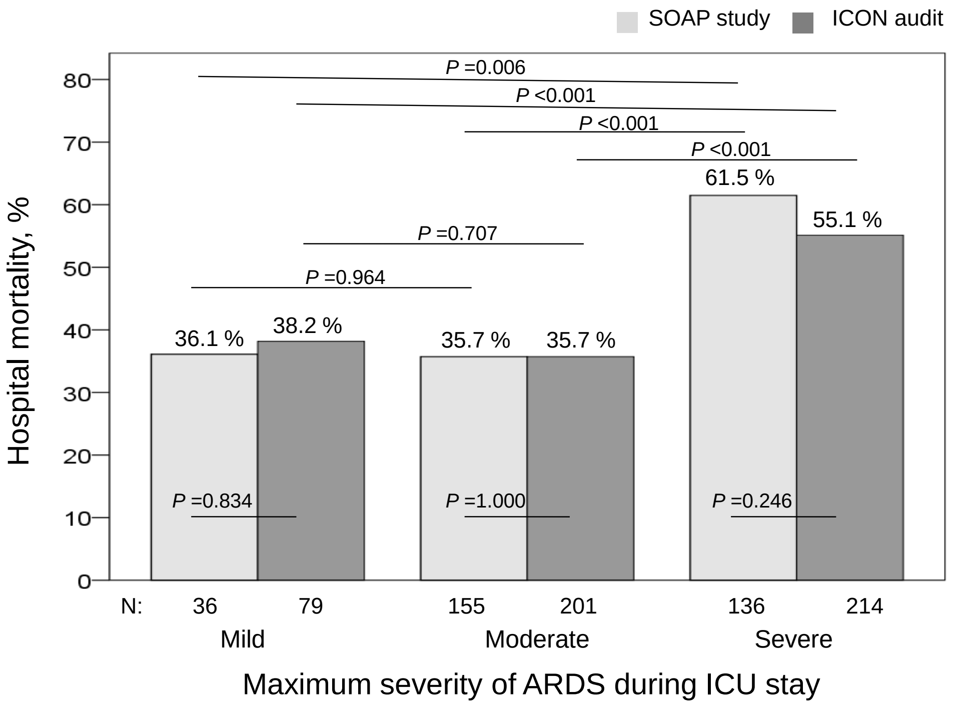

Supplement: Supplementary file 1 — Additional file 1. Supplementary tables and figure. [file 13054_2020_3455_MOESM1_ESM.pdf]
